# Low dose statins improve prognosis of ischemic stroke patients with intravenous thrombolysis

**DOI:** 10.1186/s12883-021-02259-9

**Published:** 2021-06-09

**Authors:** Chaohua Cui, Yanbo Li, Jiajia Bao, Shuju Dong, Lijie Gao, Li He

**Affiliations:** Department of Neurology, West China Hospital, Sichuan University, Chengdu, China

**Keywords:** Stroke, Statins, Intravenous thrombolysis, Efficacy and safety outcome

## Abstract

**Background:**

For acute ischaemic stroke patients, it is uncertain whether intravenous thrombolysis combined with statins might increase the therapeutic effect. Additionally, using high-intensity statins after thrombolysis may increase the risk of bleeding in patients. Asian stroke patients often take low-dose statins. It is speculated that reducing the dose of statins may improve the risk of bleeding.

**Methods:**

Data from consecutive acute ischaemic stroke patients with intravenous thrombolysis were prospectively collected. Efficacy outcomes included NIHSS (National Institutes of Health Stroke Scale) score improvement at 7 days after admission and mRS (Modified Rankin Scale) improvement at 90 days. Safety outcomes included haemorrhage events (intracerebral haemorrhage and gastrointestinal haemorrhage) in the hospital and death events within 2 years.

**Results:**

The study finally included 215 patients. The statin group had a higher percentage of NIHSS improvement at 7 days (*p* < 0.001) and a higher percentage of a favourable functional outcome (FFO, mRS <  = 2) (*p* < 0.001) at 90 days. The statin group had a lower percentage of intracerebral haemorrhage (*p* < 0.001) and gastrointestinal haemorrhage (*p* = 0.003) in the hospital and a lower percentage of death events (*p* < 0.001) within 2 years. Logistic regression indicated that statin use was significantly related to NIHSS improvement (OR = 4.697, *p* < 0.001), a lower percentage of intracerebral haemorrhage (OR = 0.372, *p* = 0.049) and gastrointestinal haemorrhage (OR = 0.023, *p* = 0.016), and a lower percentage of death events (OR = 0.072, *p* < 0.001).

**Conclusion:**

For acute ischaemic stroke patients after intravenous thrombolysis, the use of low-dose statins was related to NIHSS improvement at 7 days and inversely related to haemorrhage events in the hospital and death events within 2 years, especially for moderate stroke or noncardioembolic stroke patients.

**Supplementary Information:**

The online version contains supplementary material available at 10.1186/s12883-021-02259-9.

## Background

For acute ischaemic stroke within the time windows, IV thrombolysis (intravenous thrombolysis) is a clinically effective and guideline-recommended therapeutic method [1]. After intravenous thrombolysis, statins are regularly used as medicine for ischaemic stroke patients during hospitalization [2].

However, several studies have shown different conclusions regarding whether intravenous thrombolysis combined with statins is related to functional outcomes in ischaemic stroke patients [3–5]. Several meta-analyses have suggested that using statins in combination with thrombolysis had no significant effects on the prognosis of ischaemic stroke patients or that the combination increased the rate of haemorrhage [6–8]. Therefore, it is uncertain whether intravenous thrombolysis combined with statins is effective for ischaemic stroke patients.

A study showed that high-dose statins could increase the risk of intracerebral haemorrhage in ischaemic stroke patients [9]. Other studies have suggested that high-intensity statins after thrombolysis may increase the risk of bleeding in ischaemic stroke patients [10, 11]. These findings further raise doubts about the safety of intravenous thrombolysis combined with statins. In these studies, the patients were mainly from Europe and America, and these stroke patients mainly took high-dose statins. However, Asian stroke patients often take low-dose statins. Therefore, the conclusions may not be suitable for Asian patients. Therefore, it is speculated that reducing the dose of statins may improve the risk of bleeding in stroke patients undergoing thrombolysis.

The present prospective observational cohort study aimed to further explore the relationship between using low-dose statins combined with intravenous thrombolysis and the effects and safety outcomes of Asian ischaemic stroke patients.

## Methods

### Participants

We consecutively recruited patients with acute ischaemic stroke who received IV thrombolysis in the Neurology Department of West China Hospital, Sichuan University. The stroke diagnosis of the patients met the WHO stroke diagnostic criteria. All patients had a stroke pack (head CT: CTA + CTP) examination. The patients were enrolled from November 1, 2018, to September 30, 2020 and followed up to December 31, 2020. The study is a prospective observational cohort design.

The patients receiving statins after onset were assigned to the low-dose statin group, and the other patients who did not receive statins after onset were assigned to the control group. Low-dose statins were defined as atorvastatin 20 mg, simvastatin 10 mg and rosuvastatin 10 mg daily after onset [12]. A total of 220 patients were needed to show a significant difference [5].

The inclusion criteria were as follows: (1) age over 18 years; (2) the diagnosis of stroke with evidence of neuroimaging (CT or MRI); (3) The patients did not receive statins before onset; (4) The patients in the statin group started statins after onset for 7 days or more and (5) IV thrombolysis therapy administration within 4.5 h after stroke onset.

The exclusion criteria were as follows: (1) mRS was ≥ 2 before onset; (2) the time between admission and onset was more than 4.5 h; (3) treatment with other doses of statins after the onset of the disease or the use of statins for less than 7 days; (4) stroke associated with trauma or surgery; and (5) intracerebral haemorrhage, subarachnoid haemorrhage, coagulopathy, cancer, cardiac failure, or severe hepatic or renal dysfunction.

The study was performed in accordance with the Declaration of Helsinki and the ethical standards of the institutional and/or national research committee. The study was approved by the Ethics Committee of West China Hospital, Sichuan University with approval number 2019 (319).

### Definition of risk factors

Cardioembolic stroke was proven by definite history, electrocardiography, and cardiac colour ultrasound. The haemorrhage events included intracerebral haemorrhage and gastrointestinal haemorrhage. ICH (intracerebral haemorrhage) was proven by CT in the hospital regardless of whether the condition of the patient changed. Gastrointestinal haemorrhage included haematemesis and haematochezia. An mRS score of 0–2 was defined as a favourable functional outcome (FFO) at 90 days. A difference in NIHSS score greater than 4 was defined as an improvement. A NIHSS score of 0–4 was defined as a mild stroke, a NIHSS score of 5–15 was defined as a moderate stroke, and a NIHSS score of 16–40 was defined as a severe stroke.

### Data collection and outcome

The baseline data were collected from electronic clinical records, and structured questionnaires were completed by patients or their relatives at the patients’ admission to the hospital. The data included age, sex, blood pressure, history of smoking and drinking, NIHSS score and mRS score at admission, history of diseases and history of drug use. Types of strokes and types of statins were also collected. We recorded laboratory data, including TC (total cholesterol), TG (triglyceride), HDL-C (high-density lipoprotein cholesterol), and LDL-C. at the time of hospital admission.

The efficacy outcome was the difference in the NIHSS score between admission and 7 days after admission and FFO (mRS <  = 2) at 90 days after onset. The safety outcomes were intracerebral haemorrhage and gastrointestinal haemorrhage in the hospital and death events at 90 days after onset. We collected the NIHSS scores and in the hospital via face-to-face interviews. We collected the haemorrhage in the hospital via electronic clinical records. We collected the mRS scores and death events at 90 days stroke onset within 2 years via telephone (more than two different numbers of patients or relatives), WeChat (an instant messaging application) or e-mail. The two experienced neurologists who evaluated events and outcomes were blind to the patients’ condition and grouping.

### Statistical analysis

To describe the baseline characteristics, the continuous variables were expressed as the mean and SD (standard deviation) or median and frequencies. Categorized data and ranked data are expressed as numbers and percentages. To compare the group differences, variance analysis was conducted for continuous data following the normal distribution. A nonparametric test (Mann–Whitney U test) was conducted for continuous data that did not follow a normal distribution. A chi-square test was conducted for categorical data and ranked data.

To analyse the outcome events, we compared the group differences using the chi-square test. We analysed the effects of risk factors on the outcome events using univariable and multivariable logistic regression methods. The eligible factors of multivariable logistic regression included (1) P of univariable logistic regression less than 0.1 and (2) a factor with clinical significance. We calculated the odds ratios (ORs), 95% CIs and p values using logistic regression methods. We compared the death events at different times with a Kaplan–Meier curve. We performed subgroup analysis via univariable logistic regression by differences in NIHSS scores at admission and differences in stroke type (cardioembolic stroke and noncardioembolic stroke). The threshold for statistical significance was set as *P* < 0.05. SPSS 23.0 for Windows was used to process the data.

## Results

### Patients

The study recruited 236 patients. We lost 21 patients’ data for no follow-up or withdrew the study, and finally, the data of 215 patients were acquired.

Among the 215 patients (mean age: 70.92 ± 12.458 years)*,* 113 (52.6%) were female. The median and interquartile ranges of the NIHSS at admission were 8 (5–14). Comparing the baseline data of the different groups, the low-dose statin groups had a higher percentage of antiplatelet drug use in the hospital, a lower percentage of cardioembolic in the hospital, and lower NIHSS scores at admission than the control group. No significant difference in other baseline data was found between the two groups (Table 1).

**Table 1:** Baseline characteristic and outcome data

Variables	Low dose statin group(*N* = 180)	Control group (*N* = 35)	P*
Age, years	70.67(12.055)	72.20(14.485)	0.508
Female,%	95(52.8)	18(51.4)	0.884
Admission NIHSS score	8(4–14)	15(9–18)	** < 0.001**
Antiplatelet Drug In Hospital,%	160(88.9)	8(22.9)	** < 0.001**
Cardioembolic,%	63(35.0)	20(57.1)	**0.014**
Anticoagulation In Hospital,%	23(12.8)	7(20.0)	0.286
Systolic Blood Pressure, mmHg	148.51(24.653)	153.17(32.198)	0.333
Diastolic Blood Pressure, mmHg	84.44(16.269)	84.91(17.382)	0.876
Smoking,%	59(32.8)	10(28.6)	0.696
Hypertension,%	85(47.2)	18(51.4)	0.713
Diabetes Mellitus,%	34(18.9)	8(22.9)	0.642
Coronary heart disease,%	22(12.2)	4(11.4)	0.894
Atrial fibrillation,%	46(25.6)	17(48.6)	**0.006**
Antiplatelet drug,%	22 (12.2)	2(5.7)	0.827
Antihypertensive,%	63(35.0)	15(42.9)	0.514
Hypoglycaemic,%	24(15.3)	6(17.1)	0.716
Anticoagulation,%	12(6.7)	4(11.4)	0.394
Platelet, mmol/l	180.63(52.683)	173.91(58.295)	0.499
INR	0.98(0.106)	0.99(0.110)	0.508
ALT, mmol/l	21.56(11.390)	22.14(12.910)	0.785
Creatinine, mmol/l	77.84(26.317)	82.51(28.485)	0.344
Glucose, mmol/l	7.82(2.619)	7.99(3.447)	0.728
Triglyceride, mmol/l	1.72(1.388)	1.57(0.993)	0.544
Total cholesterol, mmol/l	4.52(1.040)	4.23(0.895)	0.123
HDL-C, mmol/l	1.30(0.396)	1.30(0.444)	0.956
LDL-C, mmol/l	2.67(0.908)	2.45(0.661)	0.095
NIHSS improvement,%	15(42.9)	145(80.6)	** < 0.001**
FFO,%	15(42.9)	121(67.2)	** < 0.001**
ICH,%	15(42.9)	19(10.6)	** < 0.001**
Gastrointestinal Haemorrhage,%	4(11.4)	1(0.6)	**0.003**
Death within 2 years,%	24(68.6)	15(8.3)	** < 0.001**

P* was calculated by ANOVA, Chi-square test, or Mann–Whitney U test as appropriate.* INR* International normalized ratio, *ALT* Glutamic-pyruvic transaminase, *HDL-C* High-density lipoprotein cholesterol, *LDL-C* Low-density lipoprotein

### Efficacy outcome

We found that the low-dose (*p* < 0.001) statin groups had a higher percentage of NIHSS improvement at 7 days than the control group. The low-dose (*p* < 0.001) statin groups had a higher percentage of FFOs at 90 days than the control group (Table 1).

In the univariate logistic regression analysis, we found that the use of statins (OR = 5.524, *P* < 0.001) and the use of antiplatelet drugs in the hospital (OR = 1.455, *P* = 0.032) were related to a higher percentage of NIHSS score improvement at 7 days after admission, and the history of hypertension (OR = 0.468, *P* = 0.018) and ICH in the hospital (OR = 0.266, *P* = 0.001) were inversely related to a higher percentage of NIHSS score improvement at 7 days after admission (Supplementary Table 1). Using statins (OR = 2.734, *P* = 0.008) and the use of antiplatelet drugs in hospitals (OR = 1.512, *P* = 0.008) were associated with FFO at 90 days. Higher NIHSS at admission (OR = 0.948, *P* = 0.011), higher NIHSS at 7 days after admission (OR = 0.938, *P* < 0.001), older age (OR = 0.975, *P* = 0.039), and higher systolic were inversely related to pressure (OR = 0.985, *P* = 0.007), and ICH in the hospital (OR = 0.452, *P* = 0.036) was inversely related to FFO at 90 days (Supplementary Table 2).

In the multivariable logistic regression analysis, after adjusting for risk factors, we found that the use of statins (OR = 4.697, *P* = 0.001) was still related to a higher percentage of NIHSS score improvement at 7 days after admission, and the history of hypertension (OR = 0.430, *P* = 0.015) and ICH in the hospital (OR = 0.367, *P* = 0.024) were still inversely related to a higher percentage of NIHSS score improvement at 7 days after admission (Table 2). Higher NIHSS at 7 days after admission (OR = 0.955, *P* = 0.022) and higher systolic pressure (OR = 0.986, *P* = 0.026) were still significantly inversely related to FFO at 90 days (Table 2).

**Table 2:** Multivariate logistic regression analysis results of efficacy outcome

Risk factor	NIHSS improvement at 7 days		FFO at 90 days	
	OR(95%CI)	P*	OR(95%CI)	P*
Using low dose statins	4.697(1.886–11.697)	** < 0.001**	1.014(0.384–2.677)	0.977
Using antiplatelet	0.924(0.627–1.362)	0.691	1.185(0.827–1.697)	0.355
ICH	0.367(0.153–0.877)	**0.024**	0.620(0.261–1.475)	0.280
History of hypertension	0.430(0.218–0.848)	**0.015**	-	**-**
Age	-	**-**	0.988(0.962–1.014)	0.359
NIHSS at admission	-	-	0.986(0.938–1.036)	0.577
NIHSS at 7 days	-	-	0.955(0.918–0.993)	**0.022**
SBP	-	-	0.986(0.974–0.998)	**0.026**

P* was calculated by multivariate logistic regression analysis, *ICH* Intracerebral haemorrhage, *SBP* Systolic blood pressure, *DBP* Diastolic blood pressure

### Safety outcome

When we evaluated haemorrhage events in the hospital, we found that the low-dose statin groups had a lower percentage of ICH events (*p* < 0.001) and gastrointestinal haemorrhage(*p* = 0.003) than the control group (Table 1). Regarding death events at the 2-year follow-up, we found that the low-dose (*p* < 0.001) statin groups had a lower percentage than the control group (Table 1).

In the univariate logistic regression analysis, we found that the use of statins (OR = 0.157, *P* < 0.001), use of antiplatelet drugs in the hospital (OR = 0.287, *P* < 0.001) and higher value of platelets (OR = 0.988, *P* = 0.008) were inversely related to a higher percentage of ICH in the hospital (Supplementary Table 2). Cardioembolic stroke (OR = 2.676, *P* = 0.010) was related to a higher percentage of ICH in the hospital (Supplementary Table 2). Using statins (OR = 0.043, *P* = 0.006) and a higher value of TC (OR = 0.379, *P* = 0.048) were inversely related to a higher percentage of gastrointestinal haemorrhage in the hospital (Supplementary Table 2). Older age (OR = 1.192, *P *= 0.019), a higher value of platelets (OR = 1.014, *P* = 0.048) and a higher value of blood glucose (OR = 1.217, *P* = 0.020) were positively related to a higher percentage of gastrointestinal haemorrhage in the hospital (Supplementary Table 2). We also found that the use of statins (OR = 0.042, *P* < 0.001) and the use of antiplatelet drugs in hospitals (OR = 0.302, *P* < 0.001) were inversely related to a higher percentage of death events within 2 years. Older age (OR = 1.061, *P *= 0.002), higher NIHSS at admission (OR = 1.122, *P* < 0.001), higher NIHSS at 7 days after admission (OR = 1.130, *P* < 0.001) and cardioembolic status (OR = 2.779, *P* = 0.005) were positively related to a higher percentage of death events within 2 years (Supplementary Table 2).

In the multivariable logistic regression analysis, after adjusting for risk factors, we found that the use of statins (OR = 0.372, *P* = 0.049), use of antiplatelet drugs in the hospital (OR = 0.414, *P* = 0.006) and higher value of platelets (OR = 0.988, *P* = 0.010) were still inversely related to a higher percentage of intracerebral haemorrhage in the hospital (Table 3). Using statins (OR = 0.023, *P* = 0.016) was still inversely related to a higher percentage of gastrointestinal haemorrhage in the hospital. Older age (OR = 1.429, *P* = 0.022) and a higher value of blood glucose (OR = 1.407, *P* = 0.026) were still positively related to a higher percentage of gastrointestinal haemorrhage in the hospital (Table 3). Using statins (OR = 0.072, *P* < 0.001) was still inversely associated with a higher percentage of death events within 2 years. Older age (OR = 1.061, *P* = 0.009) and higher NIHSS at 7 days after admission (OR = 1.075, *P* = 0.006) were still positively related to a higher percentage of death events at 2 years (Table 4).

**Table 3:** Multivariate logistic regression analysis results of safety outcome(1)

Risk factor	ICH		Gastrointestinal haemorrhage	
	OR(95%CI)	P*	OR(95%CI)	P*
Using low dose statins	0.372(0.139–0.995)	**0.049**	0.023(0.001–0.495)	**0.016**
Using antiplatelet	0.414(0.221–0.774)	**0.006**	-	-
Cardioembolic	1.974(0.863–4.513)	0.107	-	-
Platelet	0.988(0.979–0.997)	**0.010**	1.015(0.992–1.038)	0.203
Age	-	**-**	1.429(1.053–1.938)	**0.022**
Blood Glucose	-	-	1.407(1.041–1.901)	**0.026**
TC	-	-	0.061(0.004–1.061)	0.055

P* was calculated by multivariate logistic regression analysis, *TC* Total cholesterol

**Table 4:** Multivariate logistic regression analysis results of safety outcome(2)

Risk factor	Death Events	
	OR(95%CI)	P*
Using low dose statins	0.072(0.022–0.233)	** < 0.001**
Using antiplatelet	0.987(0.542–1.797)	0.965
cardioembolic	1.603(0.585–4.396)	0.359
older	1.061(1.015–1.110)	**0.009**
NIHSS at admission	1.049(0.973–1.132)	0.212
NIHSS at 7 days	1.075(1.021–1.132)	**0.006**

P* was calculated by multivariate logistic regression analysis

**Fig. 1:**
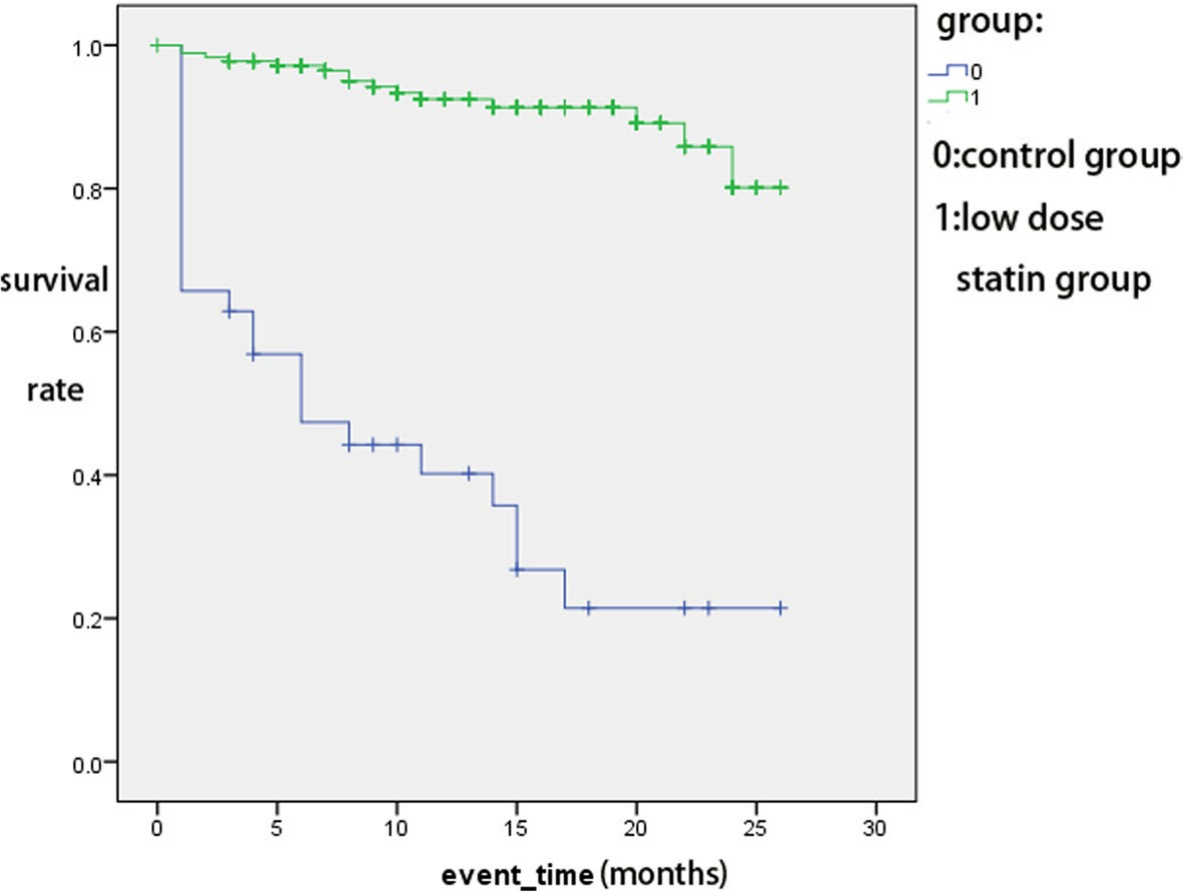
Death events by Kaplan–Meier curve

When we analysed death events in the different groups at different times with Kaplan–Meier curves, we found that the low-dose statin groups had a significantly higher survival rate than the control group at different times (Fig. 1).

### Subgroup analysis

**Fig. 2:**
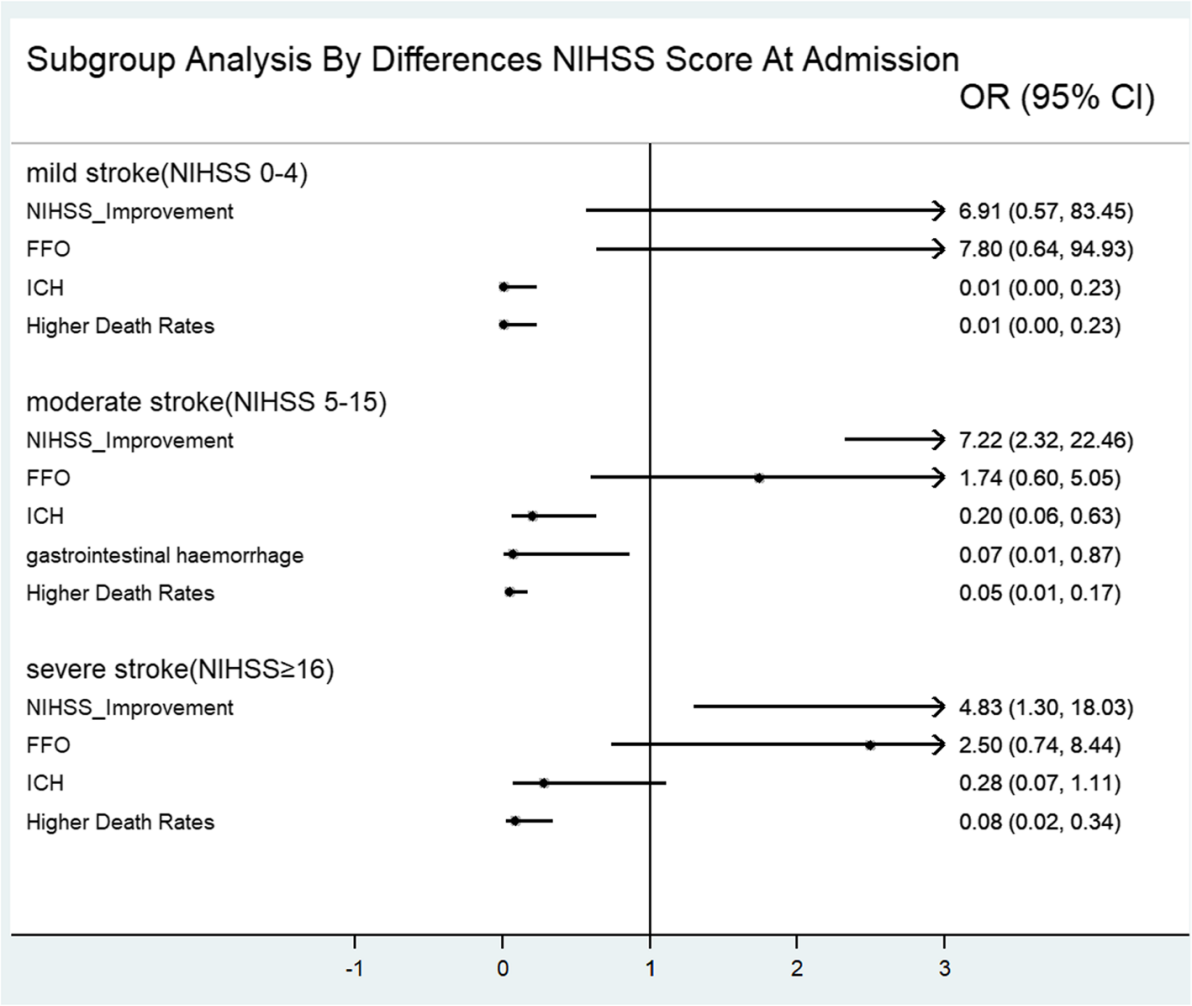
Subgroup analysis by differences NIHSS scores at admission

**Fig. 3:**
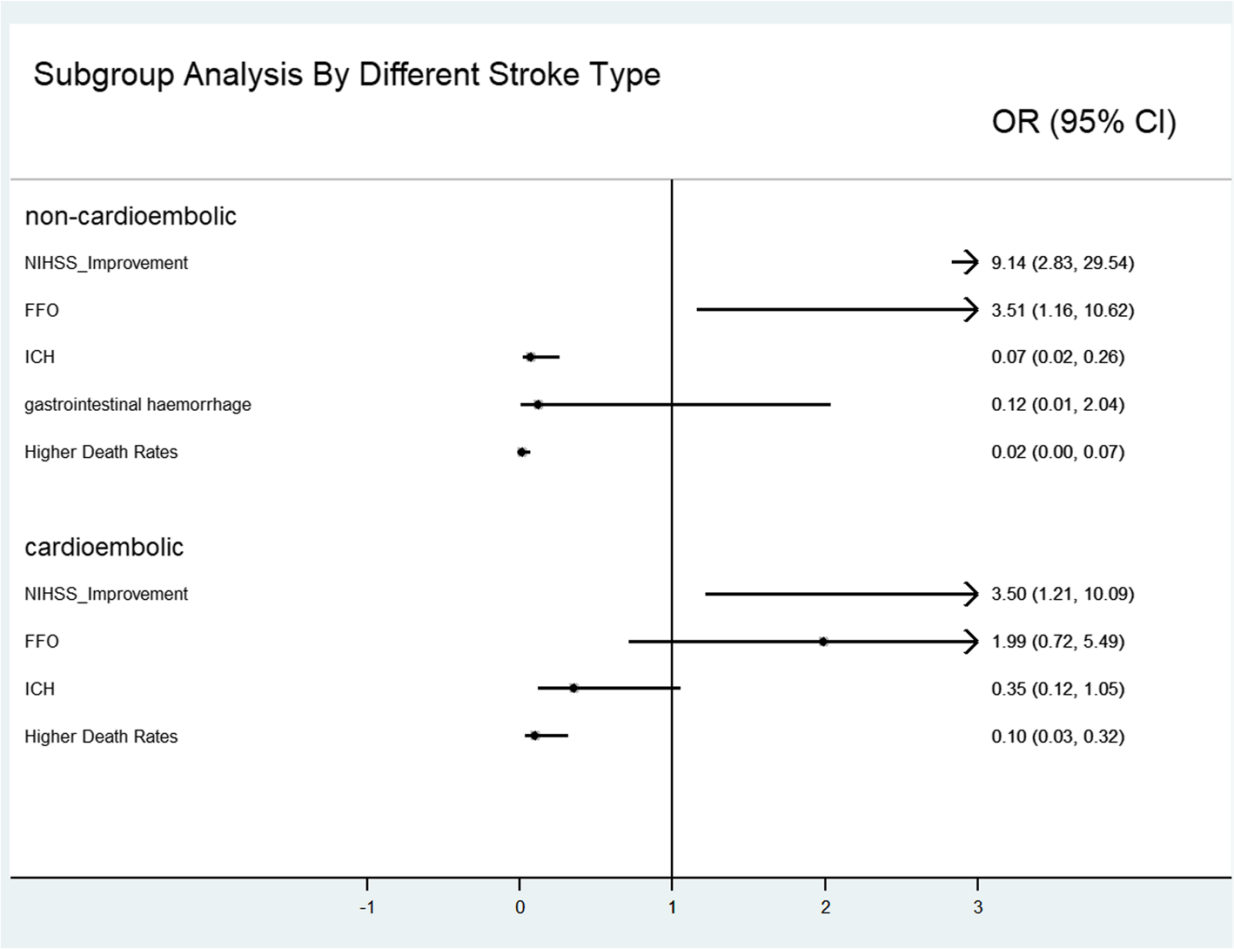
Subgroup analysis by different stroke types

In the subgroup analysis, in mild stroke patients, statin use was only significantly inversely related to ICH (OR = 0.01) in the hospital and death event rates (OR = 0.01) at 2 years. Using statins were not related to other outcomes. In moderate stroke patients, using statins was significantly related to NIHSS improvement (OR = 7.222), ICH (OR = 0.201) and gastrointestinal haemorrhage (OR = 0.074) in the hospital and death event rates (OR = 0.047) at 2 years. Using statins was not related to FFO at 90 days. In severe stroke patients, statin use was significantly related to NIHSS improvement (OR = 4.833) and death event rates (OR = 0.083) at 2 years. Using statins was not related to ICH or gastrointestinal haemorrhage in the hospital or FFO at 90 days (Fig. 2). In the cardioembolic stroke subgroup, statin use was significantly related to NIHSS improvement (OR = 3.5) and death event rates (OR = 0.102) at 2 years. Using statins was not related to ICH in the hospital or FFO at 90 days. In the noncardioembolic stroke subgroup, statin use was not related to NIHSS improvement (OR = 9.143) or FFO at 90 days (OR = 3.514). Statin use was significantly inversely related to ICH (OR = 0.353) in the hospital and death event rates (OR = 0.102) at 2 years. Using statins was not related to gastrointestinal haemorrhage in the hospital (Fig. 3).

## Discussion

We found that the low-dose statin groups had a higher percentage of NIHSS improvement at 7 days after admission and FFO at 90 days. The low-dose statin groups had a lower percentage of intracerebral haemorrhage and gastrointestinal haemorrhage in the hospital and a lower percentage of death events within 2 years than the control group. When we analysed risk factors through logistic regression, statins were significantly related to NIHSS improvement, fewer haemorrhage events in the hospital and death events within 2 years. When we evaluated death events through the K-M curve, we found that the low-dose statin groups had a higher survival rate than the control group in both 2 years. The effect of statins was more significant for moderate stroke patients or noncardioembolic stroke patients.

When the efficacy outcomes in the hospital were evaluated, our results were similar to those of Manuel Cappellari’s study [5]. The effect was significant in the different subgroups. Acute ischaemic patients after intravenous thrombolysis taking statins might have a short-term benefit for patients. When evaluating functional outcomes at 90 days, our results showed that using statins was not significantly related to FFO. The relationship in the difference subgroup had a similar result. The results were consistent with those of the low-dose statin subgroup of Manuel Cappellari’s study for 3 months of FFO [5]. However, the noncardioembolic stroke subgroup showed a significant difference. Therefore, low-dose statins might improve FFO at 90 days in noncardioembolic stroke patients after intravenous thrombolysis. We found that a higher value of SBP at admission was a risk factor for higher mRS at 90 days, and the results were not entirely consistent with Anderson’s study [13]. Anderson’s intervention was intensive blood pressure reduction, and our data were SBP. The different data types could partly explain the different conclusions. The results suggest that blood pressure management at admission plays a role in the prognosis of ischaemic stroke patients with intravenous thrombolysis.

When we evaluated safety outcomes, we found that low-dose statins were related to fewer intracerebral haemorrhage and gastrointestinal haemorrhage events through the chi-square test and logistic regression. In the mild and moderate stroke subgroups and noncardioembolic subgroups, statin use was significantly inversely related to ICH. It suggested the results had a statistic power. Our results showed that antiplatelet drug use and higher platelet counts were also related to fewer intracerebral haemorrhage events. The relationship between statin use and antiplatelet therapy was not consistent with that found in other studies [14, 15]. Patients not taking these drugs when they have an intracerebral haemorrhage might partly explain the inconsistent results. It is easy to understand the effect of platelets on intracerebral haemorrhage. The results of reduced gastrointestinal haemorrhage were consistent with a study that included myocardial infarction patients [16]. This finding suggested that statins might be a protective factor against gastrointestinal haemorrhage in acute ischaemic stroke patients with intravenous thrombolysis. However, for the different subgroups, the relationship was not statistically significant. Therefore, further study is needed to confirm this conclusion. In addition, our results suggested that increasing age and higher blood glucose levels were related to more gastrointestinal haemorrhage. This suggests that we should consider these factors when evaluating the risk of intravenous thrombolysis for acute ischaemic stroke patients.

When evaluating death outcomes, we found that using statins resulted in fewer deaths for acute ischaemic stroke patients with intravenous thrombolysis. The effect was significant for different subgroups of patients. The results were similar to Manuel Cappellari’s study about the effect of statins on the chance of death in acute ischaemic stroke patients with intravenous thrombolysis over 3 months [5]. Our results suggested that using statins to decrease death events had a longer-term effect. Within 2 years, the low-dose statin groups had significantly fewer deaths than the control group. This result suggested that low-dose statins might decrease long-term mortality for acute ischaemic stroke patients with intravenous thrombolysis. Nonetheless, more research is needed to confirm this conclusion.

Our study has several limitations. First, the study could not identify a causal relationship given the observational cohort design. However, the conditions of the prospective observational cohort were consistent with those of the real world, and the conclusions might be suitable for use in clinical practice. Second, we did not compare the specific doses of statins in our study because high-intensity statins are rarely used in Asian clinical practice, so it is difficult to obtain relevant data. Finally, the smaller number of patients in the control groups might have affected the statistical power of the results. Although we used logistic regression to increase the statistical power of the results, a study including more control group patients could further support the conclusions of the present study.

In conclusion, for acute ischaemic patients after intravenous thrombolysis, the use of low-dose statins was significantly related to NIHSS improvement, fewer haemorrhage events in the hospital and death events within 2 years. These relationships were significant, especially for moderate stroke patients and noncardioembolic stroke patients.

## Supplementary Information

**Additional file 1.**

## Data Availability

The datasets used or analysed during the current study are available from the corresponding author on reasonable request.
