# Supplementary material for: Low dose statins improve prognosis of ischemic stroke patients with intravenous thrombolysis
Source: BMC Neurol. 2021 Jun 9;21:220. doi: 10.1186/s12883-021-02259-9 (PMC8188651; doi:10.1186/s12883-021-02259-9)
Supplement: Supplementary file 1 — Additional file 1. [file 12883_2021_2259_MOESM1_ESM.docx]

**Title page:**

Title: Low dose statins improve prognosis of ischemic stroke patients with intravenous thrombolysis

Running title: Low dose statin and thrombolysis

Chaohua Cui PhD, Yanbo Li, PhD, Jiajia Bao MD, Shuju Dong, PhD,Lijie Gao PhD, Li He PhD^a^

Chaohua Cui and Yanbo Li contributed equally to this article

Department of Neurology, West China Hospital, Sichuan University, Chengdu, China

^a^Correspondence Author: Li He

Address: Department of Neurology, West China Hospital, Sichuan University, Chengdu, China

E-mail：[heli2003new@126.com](mailto:heli2003new@126.com)

**supplementary—table 1:** Univariate logistic regression analysis results

of efficacy outcome

| Risk factor | NIHSS improvement at 7 days | | FFO at 90 days | | | |
| --- | --- | --- | --- | --- | --- | --- |
|  | OR(95%CI) | P* | OR(95%CI) | | P* | |
| Using low dose statins | 5.524(2.572-11.864) | **<0.001** | 2.734(1.307-5.721) | **0.008** | |  |
| Using antiplatelet | 1.455(1.034-2.049) | **0.032** | 1.512(1.113-2.055) | **0.008** | |  |
| Using anticoagulation | 0.643(0.335-1.233) | 0.184 | 0.725(0.332-1.586) | 0.421 | |  |
| Age | 0.991(0.966-1.016) | 0.472 | 0.975(0.952-0.999) | **0.039** | |  |
| Female | 0.991(0.536-1.830) | 0.977 | 0.591(0.337-1.038) | 0.067 | |  |
| SBP | 0.991(0.979-1.003) | 0.138 | 0.985(0.974-0.996) | **0.007** | |  |
| DBP | 0.996(0.978-1.015) | 0.685 | 0.990(0.973-1.007) | 0.240 | |  |
| NIHSS at admission | 0.985(0.942-1.029) | 0.491 | 0.948(0.910-0.988) | **0.011** | |  |
| NIHSS at 7 days | - | - | 0.938(0.909-0.968) | **<0.001** | |  |
| Cardioembolic | 0.755(0.404-1.406) | 0.375 | 0.686(0.389-1.208) | 0.192 | |  |
| Smoking | 1.358(0.690-2.676) | 0.376 | 1.836(0.985-3.422) | 0.056 | |  |
| ICH | 0.266(0.124-0.569) | **0.001** | 0.452(0.215-0.948) | **0.036** | |  |
| History of diabetes mellitus | 0.541(0.263-1.116) | 0.096 | 1.057(0.532-2.134) | 0.877 | |  |
| History of hypertension | 0.468(0.250-0.877) | **0.018** | 0.988(0.567-1.721) | 0.965 | |  |
| History of CHD | 1.167(0.443-3.073) | 0.755 | 1.354(0.560-3.275) | 0.501 | |  |
| TC | 0.993(0.735-1.342) | 0.964 | 0.964(0.734-1.266) | 0.793 | |  |
| LDL-C | 0.870(0.611-1.238) | 0.438 | 0.955(0.695-1.313) | 0.777 | |  |

P* was calculated by Univariate logistic regression analysis, SBP systolic blood pressure, DBP diastolic blood pressure, ICH intracerebral haemorrhage, CHD coronary heart disease, TC Total cholesterol, LDL-C low-density lipoprotein

**supplementary—table 2:** Univariate logistic regression analysis results

of safety outcome(1)

| Risk factor | ICH | | Gastrointestinal Haemorrhage | | | |
| --- | --- | --- | --- | --- | --- | --- |
|  | OR(95%CI) | P* | OR(95%CI) | | P* | |
| Using low dose statins | 0.157(0.069-0.358) | **<0.001** | 0.043(0.005-0.400) | **0.006** | |  |
| Using antiplatelet | 0.287(0.160-0.516) | **<0.001** | 0.229(0.048-1.098) | 0.065 | |  |
| Using anticoagulation | 0.342(0.077-1.507) | 0.156 | 1.560(0.168-14.454) | 0.695 | |  |
| Age | 0.995(0.967-1.024) | 0.726 | 1.192(1.029-1.380) | **0.019** | |  |
| Female | 1.352(0.643-2.841) | 0.426 | 3.706(0.407-33.718) | 0.245 | |  |
| SBP | 0.992(0.978-1.002) | 0.262 | 1.022(0.989-1.056) | 0.191 | |  |
| DBP | 0.995(0.973-1.019) | 0.693 | 0.970(0.912-1.032) | 0.341 | |  |
| NIHSS at admission | 1.049(0.997-1.103) | 0.064 | 1.112(0.999-1.238) | 0.052 | |  |
| Cardioembolic | 2.676(1.266-5.655) | **0.010** | 2.437(0.399-14.905) | 0.335 | |  |
| Smoking | 0.862(0.387-1.918) | 0.715 | - | - | |  |
| History of diabetes mellitus | 1.940(0.845-4.452) | 0.118 | 2.833(0.458-17.523) | 0.263 | |  |
| History of hypertension | 0.960(0.461-2.001) | 0.914 | - | - | |  |
| History of CHD | 1.314(0.459-3.763) | 0.611 | 5.167(0.821-32.500) | 0.080 | |  |
| Platelet | 0.988(0.979-0.997) | **0.008** | 1.014(1.000-1.028) | **0.048** | |  |
| TC | 1.195(0.832-1.717) | 0.335 | 0.379(0.145-0.992) | **0.048** | |  |
| LDL-C | 1.205(0.789-1.839) | 0.388 | 0.389(0.136-1.117) | 0.079 | |  |
| Blood glucose | 1.940(0.845-4.452) | 0.118 | 1.217(1.031-1.437) | **0.020** | |  |

P* was calculated by Univariate logistic regression analysis, SBP systolic blood pressure, DBP diastolic blood pressure, ICH intracerebral haemorrhage, CHD coronary heart disease, TC Total cholesterol, LDL-C low-density lipoprotein

**supplementary—table 2:** Univariate logistic regression analysis results

of safety outcome(2)

| Risk factor | Death Events | |
| --- | --- | --- |
|  | OR(95%CI) | P* |
| Using low dose statins | 0.042(0.017-0.101) | **<0.001** |
| Using antiplatelet | 0.302(0.175-0.520) | **<0.001** |
| Using anticoagulation | 1.806(0.737-4.427) | 0.196 |
| Age | 1.061(1.022-1.101) | **0.002** |
| Female | 1.564(0.769-3.180) | 0.217 |
| SBP | 1.004(0.991-1.018) | 0.537 |
| DBP | 0.999(0.978-1.021) | 0.930 |
| NIHSS at admission | 1.122(1.065-1.183) | **<0.001** |
| NIHSS at 7 days | 1.130(1.086-1.176) | **<0.001** |
| Cardioembolic | 2.779(1.366-5.653) | **0.005** |
| ICH | 2.184(0.945-5.048) | 0.068 |
| Smoking | 0.487(0.211-1.124) | 0.092 |
| History of diabetes mellitus | 1.552(0.687-3.504) | 0.290 |
| History of hypertension | 1.725(0.853-3.486) | 0.129 |
| History of CHD | 2.265(0.905-5.670) | 0.081 |
| Platelet | 1.002(0.996-1.009) | 0.463 |
| TC | 0.819(0.581-1.155) | 0.256 |
| LDL-C | 0.858(0.576-1.277) | 0.450 |
| Blood glucose | 1.029(0.914-1.157) | 0.640 |

P* was calculated by Univariate logistic regression analysis, SBP systolic blood pressure, DBP diastolic blood pressure, ICH intracerebral haemorrhage, CHD coronary heart disease, TC Total cholesterol, LDL-C low-density lipoprotein
